# The role of submesoscale currents in structuring marine ecosystems

**DOI:** 10.1038/s41467-018-07059-3

**Published:** 2018-11-12

**Authors:** Marina Lévy, Peter J. S. Franks, K. Shafer Smith

**Affiliations:** 1 Sorbonne Université, Laboratoire d’Océanographie et du Climat, Institut Pierre Simon Laplace (LOCEAN, SU/CNRS/IRD/MNHN), 75252 Paris Cedex 05, France; 2 Scripps Institution of Oceanography, University of California San Diego, La Jolla, 92093 CA USA; 3 Center for Atmosphere Ocean Science, Courant Institute of Mathematical Sciences, New York University, New York, 10012 NY USA; 4 Center for Prototype Climate Modeling, New York University, PO Box 129188, Abu Dhabi, UAE

## Abstract

From microbes to large predators, there is increasing evidence that marine life is shaped by short-lived submesoscales currents that are difficult to observe, model, and explain theoretically. Whether and how these intense three-dimensional currents structure the productivity and diversity of marine ecosystems is a subject of active debate. Our synthesis of observations and models suggests that the shallow penetration of submesoscale vertical currents might limit their impact on productivity, though ecological interactions at the submesoscale may be important in structuring oceanic biodiversity.

## Abstract (web summary)

Short-lived three-dimensional submesoscale currents, responsible for swirling ocean color chlorophyll filaments, have long been thought to affect productivity. Current research suggests they may not be effective in enhancing phytoplankton growth, but may have important contributions to biodiversity.

## Introduction

**Fig. 1:**
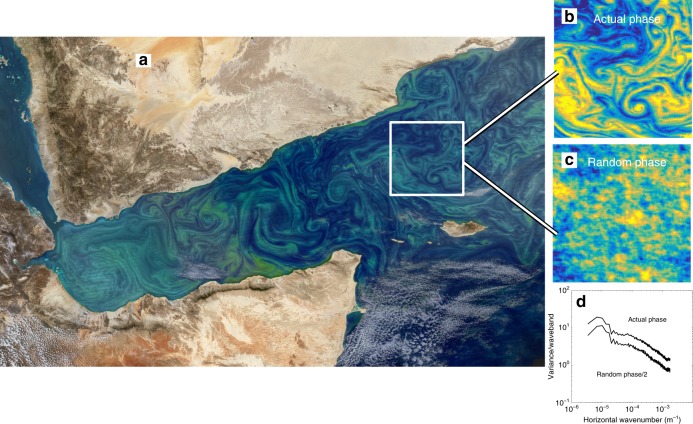
Surface submesoscale phytoplankton patchiness. **a** An image of the Gulf of Aden acquired Febuary 12, 2018 by the MODIS system on NASA’s Aqua satellite (250 m horizontal resolution), showing swirling and filamentary patterns of ocean chlorophyll (Image credit: NASA Earth Observatory) . **b** A false-color rendering of the data from the 200 × 200 km box indicated in **a**. **c** A false-color rendering of the data obtained from a two-dimensional inverse Fourier transform of the spatial spectrum from panel **b**, but with a randomized phase. The median radial spatial spectra of **b** and **c** are shown in **d**, where the spectrum from **d** has been divided by 2 to make it visible: the two spectra are statistically identical. It is clear that the coherent phytoplankton patches in **a** and **b** are a consequence of the phase relationships among the Fourier components making up the spectrum, rather than the relative magnitudes of the Fourier components (the spectral slope). Panels **b** and **c** have identical variability—their spectra are the same, but the patchiness in **b** forms coherent structures while it is random in **c**

**Fig. 2:**
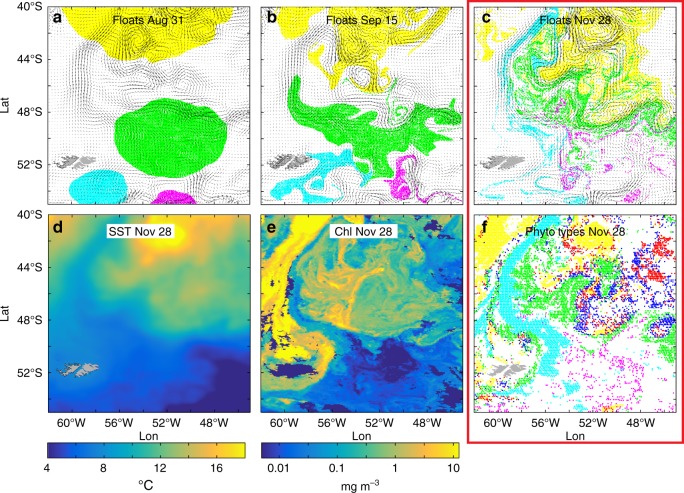
Horizontal stirring. **a**, **b** and **c** Model: virtual numerical floats advected horizontally by altimetry-derived AVISO mesoscale surface currents (arrows) from 31 August to 28 November, 2001, at the confluence of the Brazil and Malvinas currents. The currents have a nominal space–time resolution of 1/3° and one week. Floats, colored according to their original patch location on August 31, form, by November 28, small-scale tendrils through stirring by the time-evolving mesoscale flow. **d**, **e** and **f** Satellite data: **d** sea surface temperature, **e** chlorophyll, and **f** phytoplankton types. Chlorophyll data from Sea-viewing Wide Field-of-View Sensor (SeaWiFS) at 9 km resolution (**e**) were processed to derive optical products (**f**) representing dominant phytoplankton types (yellow: nanoeukaryotes; green: diatoms; magenta: *Phaeocystis*; red: *Prochlorococcus*). These types show convoluted patterns, similar to the simulated distributions of the numerical floats (**c**). The comparison of the two panels in the red box support lateral mesoscale stirring as a dominant mechanism for generating the patterns of the ecological niches in this region. Adapted from d’Ovidio et al.^48^

**Fig. 3:**
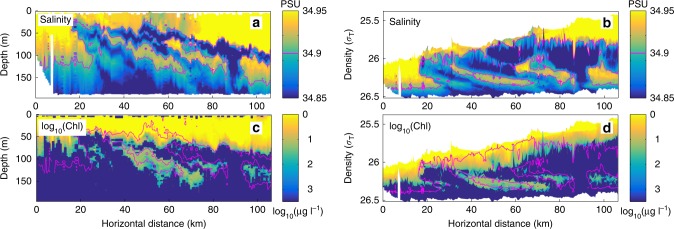
Subsurface submesoscale phytoplankton tongues. **a** Salinity and **c** chlorophyll concentration vs. depth measured along one glider transect perpendicular to the Peruvian coast, 23–29 October 2008. In **b** and **d**, the same data are plotted using density as the vertical coordinate. The magenta contour in all plots marks the 34.9 PSU salinity contour The horizontal resolution between profiles is 1 km, with 1 m vertical resolution. The section exhibits a series of interleaved tongues of high salinity/high chlorophyll water extending to 150 m depth—well below the euphotic zone. The salinity/chlorophyll tongues are ~10 km wide, and <50 m thick, forming submesoscale phytoplankton patches bounded by high spatial gradients. The fact that the layers slope across isopycnal surfaces at depth suggests that they were not formed by simple along-isopycnal subduction from the surface; other possibilities include subduction combined with surface heating, and vertical and horizontal shearing at the front. Adapted from Pietri et al.^69^

The advent of satellite radiometer measurements in 1979^1^ revealed ubiquitous swirling and filamentary patterns of ocean chlorophyll at the sea surface, with spatial scales down to the resolution of the instrument (about 4 km in 1979, and now closer to 300 m, Figs. 1 and 2). More recently, the development and deployment of fast, high-resolution sensors has begun to reveal the three-dimensional nature of these structures (Fig. 3). This filamentary patchiness extends up the trophic chain—clustering of fish, whales and seabirds near oceanic fronts, for example, has been well known to captains and fishermen for centuries^2^.

Chlorophyll is the primary pigment of phytoplankton, the microscopic free-floating organisms that can move relative to the flow by sinking, floating, or swimming, but predominantly drift passively with currents. They comprise a diverse set of organisms, with vast ranges in size, shape, and demands for their growth—the biomass and diversity of the phytoplankton community are key determinants of the structure of marine food webs. All species of phytoplankton require light and dissolved inorganic nutrients for photosynthesis. Nutrients are often scarce in the ~100 m-thick sunlit upper layer of the ocean known as the euphotic zone, but are abundant in deeper waters. Because of this vertically disjoint distribution of light and nutrients in the upper ocean, physical processes that connect the surface and interior are disproportionately important in determining the structure and dynamics of marine ecosystems. The basin-scale and seasonal distributions of light and vertical nutrient supply, for example, are fundamental determinants of marine ecological provinces^3^.

Gower et al.^1^ attributed the patchiness observed in satellite imagery to stirring of phytoplankton spatial gradients by the water motions of mesoscale eddies: vortices with lateral scales close to the internal Rossby deformation scale, which ranges from tens of kilometers in polar oceans to hundreds of kilometers in the tropics (Box 1). Later, using a simple model of two-dimensional turbulence, Abraham^4^ showed how mesoscale currents could stir the basin-scale chlorophyll gradients into thin filaments, generating scales in phytoplankton significantly smaller than those of eddies themselves—just as stirring a puddle of black and white paint creates streaks much thinner than the spoon. Importantly, stirring alone only implies the redistribution of the biological constituents, which are transported by the currents, with no change in their concentrations. Thus in principle stirring should have no consequences on the productivity or diversity of marine ecosystems.

During the last decade or so, observations and models have revealed evidence for direct forcing at scales significantly smaller than mesoscale eddies^5–7^. These submesoscale motions (Box 2) are characterized by small vortices and a plethora of rapidly changing small-scale density filaments and fronts. The lateral spatial scales of these features are in the range of a few hundred meters up to a few kilometers^8^, about an order of magnitude smaller than the latitude-dependent deformation scale. Upper-ocean submesoscale currents are three-dimensional^9^: intense along front lateral currents, combined with a secondary vertical circulation. These currents may drive nutrients from the deep pool into the euphotic zone—and drive phytoplankton into the dark. Submesoscale processes generally also reduce mixed-layer depth, increase vertical stratification, and decrease vertical mixing, with consequences for the residence time of phytoplankton in the euphotic zone. Thus, in contrast to mesoscale stirring, submesoscale forcing can affect growth rates, biomasses, biogeochemical fluxes, and community structure.

Swirling phytoplankton features are often characterized by the presence of a local maximum (of species, taxa, or types), bounded by strong horizontal concentration gradients (Figs. 1 and 3). Because at least one of the two horizontal dimensions of such features falls in the submesoscale range, they are often referred to as submesoscale and we will use this terminology throughout the paper. We make a distinction here between submesoscale patchiness and submesoscale variability. Submesoscale features, or patches, will appear as coherent blobs, streaks, and whorls of a property as shown in Fig. 1. Variability, on the other hand, is the magnitude of variation of a property at a given spatial scale.

Disentangling whether submesoscale phytoplankton patches are generated by stirring of existing biological gradients, or are an active response to submesoscale physics, is therefore key to quantifying their global impact on marine ecosystems: are they nothing more than astonishing rearrangements, or do they reflect a complex response of the ecosystem to forcings that are difficult to observe, model and quantify? Submesoscale currents are continuously forming, moving, and dissipating over time scales ranging from days to weeks, making them particularly difficult to sample and model. Because biological response time scales are long enough to allow displacement of the biological response from the proximate physical forcing, submesocale forcings are even more difficult to relate to ecosystem dynamics. Satellite altimeters, used to provide global maps of upper-ocean currents, do not presently resolve velocity features smaller than about 100 km, and so entirely miss submesoscale currents. Numerical models require extremely fine grid resolution to explicitly resolve these dynamics, which is computationally challenging. And the logistical issues of sampling and modeling short time-scale, small spatial-scale dynamics are compounded by a lack of tools to quantify biological properties—particularly rates, such as growth and grazing—in a fast-moving frame of reference.

**Fig. 4:**
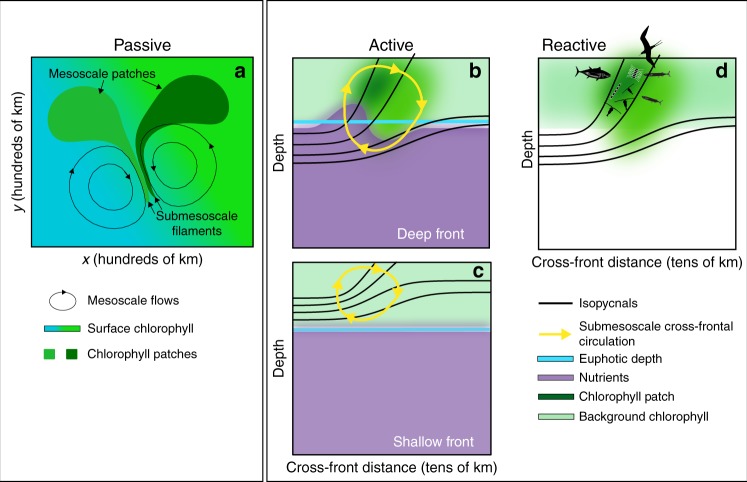
Schematic representation of passive, active, and reactive mechanisms. **a** Passive deformations of existing phytoplankton patches by mesoscale currents can form submesoscale filaments. **b** Changes in phytoplankton growth rates driven by submesoscale nutrient fluxes into the euphotic zone can lead to the formation of phytoplankton patches at deep fronts. If the submesoscale circulations do not penetrate into the nutricline below the euphotic zone at shallow fronts, then it is unlikely that phytoplankton patches will form **c**. **d** Phytoplankton reactions (e.g., growth, competition, swimming, loss by grazing, etc.) and behavioral responses of higher trophic-level organisms (including fish, birds, and mammals) in response to passive and active processes

Submesoscale phytoplankton features form through an assortment of often-concurrent, highly interconnected mechanisms that drive planktonic ecosystem reactions, affecting diversity, competition, and marine food-web structure. We propose here a conceptual framework to sort the mechanisms responsible for the formation of submesoscale patchiness, guide our synthesis of observations and high-resolution models, and focus our discussion of their biogeochemical and ecological implications. We begin by analyzing the ways in which submesoscale dynamics drive active responses in phytoplankton growth rates—the central topic of this review. For example, strong submesoscale vertical velocities may bring limiting nutrients into the euphotic zone, causing a rapid, local increase in phytoplankton growth rates (Fig. 4b). We then explore how submesoscale biological patchiness can be generated by passive processes: the stirring and mixing of existing biological features by ocean currents (Fig. 4a). This occurs with phytoplankton blooms, for example, when stirring is faster than the local biologically driven rates of change. Finally, we discuss biological processes driven by a reaction to active and passive forcings (Fig. 4d). These biological reactions can be behavioral (i.e., swimming) or ecosystem processes (i.e., changes in community structure), and may themselves lead to biological features with distinct spatial scales. This analysis leads us to discuss why submesoscale dynamics might be significant for ecosystem diversity, but less important for marine productivity.

### Box 1 A brief history of the mesoscale

A discussion of submesoscale dynamics requires some historical and conceptual understanding of the mesoscales. Up through the 1950s, basic theory considered the ocean to be characterized by gyre-scale currents and meters-scale turbulence, with little in between. Oceanic “eddies” were first inferred from the erratic patterns of John Swallow’s trackable floats^129^, and from time series of moored current arrays in the early 1960s^130^. These indicated the presence of time-variable currents with amplitudes relatively large compared to their mean values. It took another decade for a shadow of the spatial structure of these disturbances to be revealed by the USSR POLYGON experiment and the US Mid-Ocean Dynamics Experiment MODE^131^. Both of these used tightly coordinated campaigns of ship-based observations and current-meter arrays to piece together a picture of “mesoscale” dynamics; both programs focused on relatively small regions in the North Atlantic. They revealed elliptical eddies with lateral scales of about 100–200 km and time scales of months.

As first explained by Gill et al.^132^, the spatial and temporal scales of mesoscale eddies are consistent with baroclinic instability (also the dominant source of midlatitude weather systems) which converts the massive potential energy stored in the ocean’s sloping density surfaces—an energy source 1000 times as large as the kinetic energy of oceanic currents—into turbulent eddies. The dominant lateral scale of baroclinic instability is near the Rossby deformation scale, *L*_D_ = *NH*/*f*, where *N* is the buoyancy frequency, *f* is the Coriolis frequency (which vanishes at the equator and is maximum at the poles), and *H* is roughly the depth of the thermocline. This is the scale at which rotational and buoyancy effects play equally important roles in the dynamics. Typical values of *L*_D_ range from tens of kilometers in the polar oceans to hundreds of kilometers in the tropics.

Satellite altimetry, beginning with the short-lived SEASAT program in 1978, revolutionized oceanography, enabling synoptic views of entire ocean basins^133^. Altimetric measurements show the spatial patterns of sea-surface height, from which currents can be estimated via geostrophic balance: on a rotating planet horizontal pressure gradients are approximately balanced by the Coriolis force, causing currents to flow around elevated regions (clockwise around elevated regions in the Northern Hemisphere, and so forth). As technology improved, a global synoptic view of the mesoscale ocean at last emerged with the TOPEX/Poseidon program in the 1990s^134^. Observations from this satellite revealed eddies nearly everywhere (as seen, for example, in these two plots of AVISO sea-level anomaly from January 31, 2013 over the Atlantic and Pacific oceans, image credit: AVISO/CNES/CLS), with scales of order 100 km, decreasing with increasing latitude, roughly proportional to but larger than the latitudinal scaling of *L*_D_. However, ocean variability on smaller scales is difficult to infer from current satellite-based altimetric instruments, which have an effective resolution near the deformation scale—about 100 km. Features seen in satellite altimetry are intrinsically dynamic, thus the absence of such global measurements at smaller scales is a severe limitation on our ability to separate dynamic from kinematic effects in submesoscale structures.

### Box 2 Frontal and submesoscale dynamics

Submesoscale motions, with lateral scales a decade or so below the mesoscale, are best defined in terms of dynamical balances^11^: unlike mesoscale dynamics, submesoscale dynamics are not well-approximated by geostrophic balance, and so all the terms in the equations of motion are potentially important. Submesoscale dynamics are often closely linked with fronts. The purpose of this box is to give a brief overview of the physical forcings that lead to fronts and submesoscales, and the phenomenology of the flows themselves.

Early evidence of dynamical features much smaller than the mesoscale came from sun-glitter in photographs taken during the 1970s Apollo Mission, which revealed predominantly cyclonic spiral eddies in the ocean, with scales of around 10–25 km^135^. All such submesoscale motions derive their energy from lateral density gradients in the surface ocean (see schematic). These density gradients are driven by atmospheric forcing: heat and fresh-water exchanges, and winds, which inhomogeneously mix the upper ocean. Horizontal straining by mesoscale eddies (red arrows) can squeeze these density contrasts into nearly vertical planes (gray surfaces), intensifying their geostrophic along-front, sheared currents (green arrows)—this is an example of frontogenesis. Fluid instabilities feed off these localized sources of potential and kinetic energy, generating submesoscale vortices and filaments that carry of some of this energy away. A sufficiently strong front will generate a cross-frontal ageostrophic secondary circulation—an overturning circulation directed in the sense of trying to flatten the density surfaces in the front (yellow arrows). Next we describe persistent fronts, submesoscale fronts, and a more complicated scenario that inextricably links mesoscale and submesoscale motions.

Persistent fronts like those associated with the Gulf Stream and Kuroshio are locked in place by the coastal boundary and large-scale atmospheric forcing. Strong winter-time winds are directed nearly parallel to the axis of the jet, driving cold dense water southward across the front through Ekman forcing—a dynamical balance between surface friction, pressure, and Coriolis acceleration that moves upper ocean waters to the right of the wind in the Northern Hemisphere—keeping the density surfaces steep, the stratification low, and strengthening the jet^24, 136^. This forcing is directly balanced by submesoscale symmetric instability, which takes energy mostly from the kinetic energy of the jet, and baroclinic instability, which converts the potential energy of the sloping density surfaces into large meanders and eddies. Associated with these highly energetic processes is a deep vertical velocity structure^137^ that reaches into the thermocline.

The ubiquitous submesoscale density fronts and filaments that are continuously created at the ocean’s surface by mesoscale strain differ from persistent fronts like the Kuroshio and Gulf Stream. Unlike their more powerful cousins, submesoscale fronts are continuously forming, moving, and dissipating—they are not in a steady-state balance between forcing and dissipation at a fixed location. These fronts have associated cross-frontal secondary circulations that are generally confined to the vertically well-mixed upper layer of the ocean^138^. In the presence of shallow summer-time mixed layers, these features do not carry much energy and thus have little impact on the local circulation. In the winter, on the other hand, the nearly vertical density surfaces associated with these fronts and filaments extend through the deep mixed layer, and so contain significant potential energy in addition to the intense kinetic energy of their cross-front currents. In the last few years, ample evidence^8, 139–141^ indicates that this energy is converted to kinetic energy by mixed layer instability^142, 143^: a submesoscale baroclinic instability with maximal growth at the mixed-layer deformation scale, *L*_ML_ = *N*_ML_*h*_ML_/*f*, where *N*_ML_ and *h*_ML_ are, respectively, the mixed-layer buoyancy frequency and mixed-layer depth. In mid-latitudes in winter, with *N*_ML_~10^−3^ s^−1^, *h*_ML_~200 m and *f* ~2 × 10^−4^ s^−1^, this gives *L*_ML_ ~1 km, and like mesoscale baroclinic instability, the result is the formation of submesoscale vortices with sizes close to this scale^143^.

Sufficiently steep density contrasts confined to the mixed layer can generate significant submesoscale energy, as above. But less-steep density surfaces that intersect the surface and reach deep into the interior can also generate significant energy at submesoscales, through a process called Charney-type baroclinic instability, which is likely widespread in the ocean^144^. This mesoscale-driven process provides an alternate but potentially important pathway between the surface and interior^23, 25^.

In all cases, submesoscale processes act against fronts, reducing mixed-layer depth, increasing stratification, and decreasing vertical mixing^145^. The eddy kinetic energy produced in these processes likely enhances lateral turbulent mixing at the submesoscale^146^. Ultimately, submesoscale turbulent energy undergoes an inverse cascade, coagulating small vortices into larger features and ultimately increasing the energy of the mesoscale^5, 31, 147^ indirectly modifying the large-scale circulation.

## Active processes alter phytoplankton growth rates

The temporal and spatial scales of submesoscale currents are often similar to those of phytoplankton growth time scales and patch scales, suggesting the possibility of close coupling of phytoplankton growth with submesoscale forcings. One feature of submesoscale dynamics that makes them particularly relevant to planktonic ecosystems is that they drive strong local vertical velocities at fronts^9–12^ (Box 2). These vertical velocities may drive locally enhanced nutrient fluxes up into the euphotic zone, and pull phytoplankton down into the dark ocean interior.

Frontal dynamics at submesoscales is a topic of current research, and categorizing the various types of fronts is a necessary precursor to discussing the role of submesoscales in biology. Broadly, there are a range of fronts, with persistent fronts and ephemeral fronts representing the two extremes (Box 2). Persistent fronts include, for example, the intense horizontal density gradient associated with the Gulf Stream and Kuroshio (and many others), which have lateral scales on order 10–50 km, and which are relatively steady on timescales of a month or so. They are held in place by coastal boundaries and atmospheric forcing, have strong along-front currents, and large vertical velocities associated with a cross-frontal ageostrophic circulation that reaches well into the ocean’s interior. Fast-changing, smaller, ephemeral (or, simply, submesoscale) fronts and filaments form through both secondary instabilities associated with forced, persistent fronts, and in the open ocean, through the generic process of straining by mesoscale currents and open-ocean submesoscale instabilities. These fronts evolve on timescales of days to weeks, and like their larger, forced cousins, have strong cross-frontal circulations that are out of geostrophic balance. However, the associated vertical velocities are typically trapped within the ocean’s well-mixed surface layer.

### Upward nutrient supplies by submesoscale transport

In mid-latitude and high-latitude, wintertime convective mixing brings nutrients into the euphotic zone, and these are consumed when light conditions become favorable, leading to strong seasonality in phytoplankton abundance with a pronounced springtime maximum; it is in only in summer that the environment is oligotrophic, i.e. that the euphotic layer is deprived of nutrients. In contrast, the subtropics are oligotrophic “ocean deserts”—despite having plenty of light year-round, because large-scale downwelling and shallow mixed layers limit nutrient supplies from the deep. In such nutrient-depleted oligotrophic environments, phytoplankton populations respond to upward nutrient inputs into the euphotic zone by submesoscale vertical velocities on time scales of hours to days, exhibiting locally increased growth rates and subsequent increased biomass^13^. Though sampling such transient and small-scale events is difficult, new technologies such as profiling floats are providing direct evidence of short-lived, submesoscale nutrient patches, presumably indicative of vertical nutrient fluxes at the same lateral scale^14–18^. Associations between frontal vertical velocities and chlorophyll patches have been observed in high-resolution surveys conducted in frontal regions^6,19^. In these studies, vertical velocities were derived from the quasigeostrophic omega-equation, and mapped onto chlorophyll distributions at horizontal resolution ~20 km. Other studies have shown that specific submesoscale chlorophyll patches present in ocean color images were located over regions of strong stretching^20^ or divergence (and thus of upwelling)^21^, identified through Lagrangian analyses of mesoscale surface currents derived from satellite altimetry. More recently, intense submesoscale chlorophyll features in ocean color images have been statistically connected to the presence of temperature fronts in the North Pacific subtropical gyre^22^, another piece of indirect evidence for a submesoscale phytoplanktonic growth response to nutrient upwelling at submesoscale fronts.

**Fig. 5:**
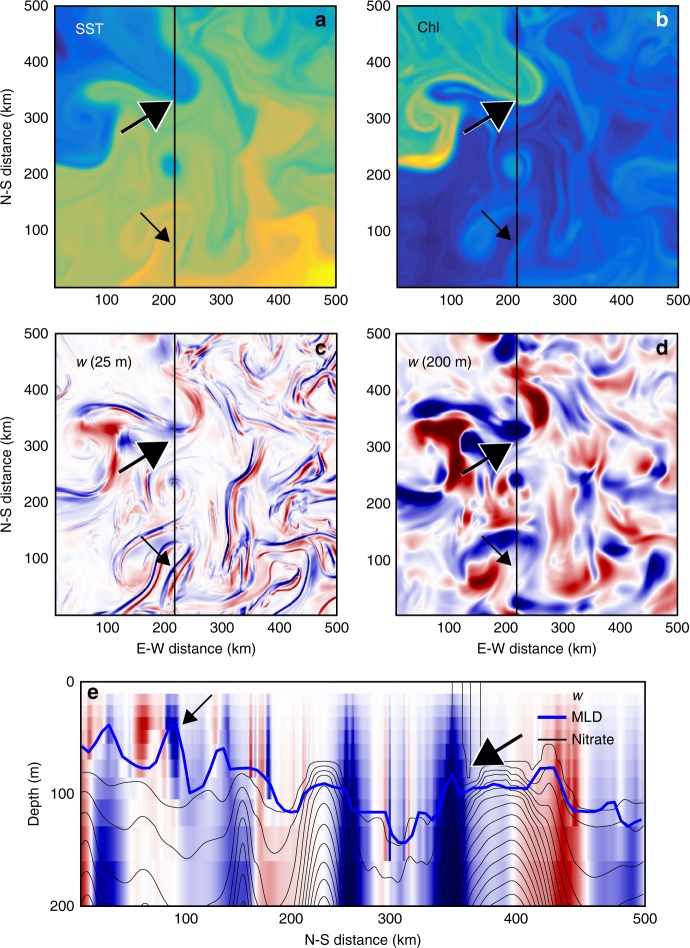
Submesoscale vertical advection at fronts. **a** Sea surface temperature (SST), **b** sea surface chlorophyll (Chl), and **c**, **d** vertical velocities (*w*: red = upwelling, blue = downwelling) **c** in the surface mixed-layer (at 25 m depth), and **d** below (at 200 m depth) in a 500 km × 500 km box from a larger ocean general circulation model representing the Gulf Stream region (the full model domain is shown in Box 3; these data come from the black square region). **e** A vertical section taken along the black line of **a**–**d** showing vertical velocity (blue-red), with nutrient contours (black lines) and mixed-layer depth (heavy blue lines) overlaid. Vertical velocities associated with the persistent front (meandering Gulf Stream, large black arrows) extend from the surface and penetrate below the nutricline (delinated as the first nutrient contour). The near-surface vertical velocity field also exhibits thin, elongated submesoscale structures, located at submesoscale temperature fronts (small black arrows). Unlike the vertical velocities associated with the persistent front, the vertical velocities at submesoscale fronts do not extend much below the surface mixed layer, and do not always reach the nutricline. This results in a response of surface phytoplankton at the deep persistent front (yellow color show maximum phytoplankton concentrations at the front, large black arrows) but not at the shallow submesoscale front (small black arrows). Adapted from Lévy et al.^26^

A key factor determining the magnitude of vertical nutrient fluxes is the depth of penetration of vertical velocities into the nutricline: vertical velocities confined to a mixed layer above the nutricline (such as those often associated with ephemeral fronts) do little besides mix the nutrient-poor surface layer^23^ (Fig. 4c). On the other hand, deep, dynamic, persistent fronts that extend into the nutricline below the mixed layer can provide a nutrient-flux pathway to the euphotic zone from the interior^24,25^, and so may be effective in altering budgets of biogeochemical tracers (Fig. 4b). This upward nutrient flux may reach the surface mixed layer to drive surface blooms, or, may only reach the base of the euphotic zone to fuel subsurface blooms^5,17^. Examples of deep and shallow fronts were examined in one submesoscale-resolving model of a generic oligotrophic subtropical gyre^26^. In this model, the vertical velocities associated with submesoscale dynamical fronts were largely confined to the surface mixed layer, contributing little to the vertical nutrient flux. In contrast, the vertical velocities associated with the quasi-permanent Gulf Stream-like front reached well below the mixed-layer and into the nutricline, driving strong vertical nutrient fluxes (Fig. 5).

Whether submesoscale-driven nutrient fluxes have a broader spatial relevance is a topic of current investigation. Biogeochemical models run with horizontal grid-cell sizes differing by two-orders of magnitude (typically from 1 to 100 km) have consistently shown that productivity increased with increased model resolution over the first months to years of model integration^5,26–30^. This could be interpreted as support for the global importance of active responses to the submesoscale dynamics resolved by the high-resolution models. However, while improved model resolution allows stronger submesoscale fronts, it also leads to stronger persistent fronts that may account for much of the enhanced nutrient flux and increase in basin-integrated productivity (Box 3). We must therefore be careful to distinguish whether model simulations with different resolutions drive changes in productivity through the emergence of submesoscale fronts, or through the strengthening of persistent fronts.

Though they act at small scales, submesoscale dynamics appear to play a role in models of the basin-scale ocean circulation and tracer distribution. For example, comparing solutions from otherwise-identical high-resolution (1/54°) and coarse-resolution (1/9°) models^26,31^ showed that after more than 50 years the models diverged, with the high-resolution solution showing a deeper nutricline and shallower winter mixed layer in the subtropical gyre. This resulted, counter-intuitively, in less productivity in the subtropics, despite more submesoscale activity. This result illustrates a basin-scale ecosystem response to submesoscale forcing (productivity decrease) in opposition to the submesoscale response (locally increased productivity).

There is also a strong seasonal component to the structure and intensity of submesoscale vertical velocities at higher latitudes: the horizontal buoyancy gradients that underlie vertical velocities at submesoscale fronts are strongest in deep wintertime mixed layers^32^. However, over vast regions of the ocean, including the subtropical gyres, temperate zones, and high latitudes, wintertime mixed layers are already replete with nutrients through convective mixing. Thus in wintertime, submesoscale vertical velocities may not be effective in driving nutrient transport to the euphotic zone. In summer, shallow mixed layers are nutrient-depleted and phytoplankton growth rates can be enhanced by vertical nutrient fluxes. However, thin surface mixed layers create weaker summer submesoscale vertical velocities that may not penetrate down to the nutricline^8,32,33^. Satellite observations of chlorophyll in the North Pacific subtropical gyre tend to confirm the strong seasonality of the impact of submesoscale dynamics on phytoplankton biomass, showing a negligible effect in summer^22^. This annual cycle suggests that over large regions of the world’s ocean, submesoscale-driven vertical transport could be out of phase with the seasonal nutrient requirements of phytoplankton.

The seasonality and shallow penetration of vertical velocities at submesoscale fronts bring into question their efficacy for upward transport of nutrients, particularly in comparison to persistent fronts. Nevertheless, modeling studies suggest that vertical transport at submesoscale fronts could be significantly enhanced in the presence of variable winds^34,35^, and that the presence or absence of high-frequency winds might be a critical factor influencing the depth reached by nutrient fluxes at fronts^36,37^. More systematic analysis is required to disentangle the relative contributions and scales of the fluxes associated with different varieties of front^38^, in presence or absence of winds.

### Box 3 Capturing submesoscale dynamics with Ocean General Circulation Models (OGCMs)

OGCMs are computer codes that estimate solutions to the oceanic primitive differential equations of fluid motion and thermodynamics discretized on a spatial grid that covers the word’s ocean. These models form the ocean core of Earth System Models (ESMs). Their computational cost depends on the number of grid points. A decrease of the horizontal grid size by a factor of *N*, equivalent to an increase in the number of grid points by a factor of *N*^2^, increases the computational cost by *N*^3^, because higher resolution also requires smaller time steps^31^. Since the 1970s when OGCMs were first developed, their horizontal grid resolution has always been constrained by the capacity of supercomputers. This capacity has increased tremendously over the last decade. While ocean grids used for the 5th Coupled Model Intercomparison Project (CMIP5) of the World Climate Research Program (WCRP) ranged between 1° and 2°, ocean grids with ~1/10° resolution are emerging in CMIP6. Mesoscales and submesoscales are thus smaller than the grid size of current ESMs and parameterizations are used to mimic their large-scale effects^143, 148^. Reducing uncertainties linked to such subgridscale processes is one of the biggest challenges in ocean modeling^149^.

Nevertheless, explicit resolution of submesoscale processes is achievable with regional or idealized models, which use the same computer codes as OGCMs but with reduced domains, allowing for finer grid cells. One example taken from Lévy et al.^150^ is reproduced here: the model equations are solved over a rectangular ocean basin of ~20° × 30° with closed boundaries. The circulation and thermodynamics are forced by seasonally varying winds and heat fluxes at the sea surface. The model equations are integrated on three different horizontal grids of resolution 1°, 1/9°, and 1/54°, and with the same vertical resolution of 30 vertical layers, 10 of which are in the upper 100 m. Common features emerged from the three model solutions including a strong persistent front (marked by the white SST contour) extending toward the northeast, separating a warm subtropical gyre from a cold subpolar gyre. Strong horizontal turbulence emerged as the resolution was increased. At 1/9°, the turbulence appeared as the meandering of the persistent front and in the formation of a few mesoscale eddies. At 1/54°, sharp submesoscale temperature fronts were present and the meandering of the persistent front was more pronounced. In addition, the persistent front was progressively displaced southward when the resolution increased. Thus, resolving small-scale ocean processes can lead to substantial changes in large-scale patterns.

Importantly, the vertical velocity field close to the ocean surface was strongly modified both in its structure and intensity as the resolution increased. While at coarse resolution the main patterns in vertical velocity were coastal upwellings with subsidence in the center of the subtropical gyre, energetic fine structures in vertical velocity became more prominent as the resolution increased. These changes in SST and vertical velocity were also reflected in their spectral slopes (red and blue curves show the spectra at 1/54° and 1/9°, respectively), with much flatter spectra at 1/54° than at 1/9° resolution.

One might question to what extent submesoscale processes are adequately represented at 1/54° resolution. One indication is provided by the power spectra, which show a sharp decrease of variance at scales smaller than 10 km (gray shading). This is the dissipative range for this model resolution: the effective model resolution is closer to 1/9° when the grid resolution is equal to 1/54°. Thus it appears that a submesoscale-resolving grid enables much better resolution of the mesoscale dynamics, providing the straining and stirring environment favorable for the emergence of submesoscale dynamics. This is clearly seen in the spatial spectra in the 10–100 km range: there is more energy with a 1/54° resolution than with a 1/9° resolution. The submesoscale dynamics, however, are still strongly damped at 1/54° resolution, which explains why this resolution range is often referred to as submesoscale permitting rather than submesoscale resolving.

Recent studies suggest that an order of magnitude increase in resolution is required to adequately capture the full strength of the submesoscale dynamics, both in the vertical^8^ and horizontal^151^ dimensions. This resolution is not yet achievable with present-day computer capacities for regional biogeochemical studies; improved understanding will continue to depend on technological progress in high-performance computing.

### Growth limitation by downward submesoscale transport

Submesoscale vertical velocities may also limit growth by moving phytoplankton and nutrients out of nutrient-rich environments^26,28,39^. The downward branch of frontal circulation cells that reach below the base of the euphotic zone may be especially effective in this regard^26,28,39^. In eastern boundary upwelling systems and equatorial regions, for example, nutrients are abundantly supplied to the euphotic zone by large-scale upwelling, sustaining elevated levels of phytoplankton growth. In such systems, model simulations support the suggestion that the observed reduction of biological production in regions of elevated eddy kinetic energy might be due to the downward leakage of both phytoplankton and nutrients from the nearshore to the open ocean^40,41^. In the North Atlantic, where seasonal productivity is characterized by the occurrence of a strong spring bloom, the observational and high-resolution modeling study of Omand et al.^42^ suggests that the most favorable conditions for efficient export are during the transition from winter to spring.

### Light exposure enhanced by submesoscale stratification

Several field and modeling studies have shown that submesoscale dynamics may also increase phytoplankton residence times in the euphotic zone of deep mixing layers^43^ by tilting existing horizontal density gradients to increase the vertical stratification of surface mixed layers^7,44,45^. This vertical-shear-driven increase in vertical stratification reduces the local turbulence; in mid and high latitudes (with most observations from the North Atlantic) the increased residence time of phytoplankton in the euphotic zone leads to early local phytoplankton blooms compared to surrounding, actively mixing waters^7,46^. The horizontal scale of such changes in vertical turbulence is set by the submesoscale circulation; this leads to submesoscale patches of enhanced phytoplankton growth rates, which can ultimately form submesoscale patches of phytoplankton earlier than the regional average. Such early, local blooms may not change the seasonal productivity budget^47^ but presumably affect the seasonal species succession and local community structure.

Finally, submesoscale vertical velocities also have the potential to penetrate into the deep chlorophyll maximum, displacing low-light-adapted cells upward and high-light-adapted cells downward, altering the community structure at the base of the euphotic zone, and potentially altering the net growth rate there^5^.

## Passive processes reorganize phytoplankton without affecting biomass

We now consider the complementary issue of the generation of submesoscale biological features through passive stirring. Stirring by larger-scale currents can readily generate horizontal concentration gradients at scales much smaller than the flow itself (Fig. 4a), complicating the attribution of submesoscale biological patches to their forcing.

### Surface stirring stretches phytoplankton into submesoscale patches

Consider an example of a few smooth mesoscale vortices a few hundred kilometers in diameter, with no dynamic features at smaller scales. A tracer patch in the surface mixed layer stirred by this current will be stretched out in one direction, and laterally compressed in the other by the strain between eddies (Fig. 4a). The thinnest width of a tracer filament will be set by a competition between the rate of compression/stretching, which thins the tracer, and the rate of horizontal diffusion, which broadens it. The resulting spatial patterns of chlorophyll will have smaller-scale variability than the flow itself.

d’Ovidio et al.^48^ (Fig. 2) used satellite altimetry to generate mesoscale surface velocity fields in an eddy-active region of the ocean. These smooth fields were used to advect virtual particles, representing the four broad groups of phytoplankton types that were identified from ocean color data in this region. The stirring stretched the initial large-scale surface patches of virtual phytoplankton types into submesoscale patches whose shapes were visually similar to those seen in satellite color and SST data, at least at the mesoscale. At submesoscales (below roughly 1/2° in Fig. 2), the correlation is more questionable. This may be due to the limited (~100 km) resolution of the underlying velocity field, or to inhomogenous biological reactions, which can alter the structure of the variance^49,50^. Similarities between the satellite data and the modeled surface phytoplankton distributions, however, suggest that stirring by smooth mesoscale currents may be a key player here, distorting the large-scale phytoplankton landscape into submesoscale patches delineated by sharp gradients.

The rearrangement of spatial gradients by stirring can sometimes be detected in the different slopes of the variance spectra (a Fourier transform of the squared spatial property anomaly) of the underlying flows and the property being stirred. Oceanic velocity power spectra often exhibit power-law behavior, with spatial variance proportional to *k*^*−a*^, where *k* is the wavenumber, and −*a* the spectral slope on a log–log plot. Flatter slopes (less negative, e.g., *k*^−1^) indicate more variance at small scales than steeper slopes (more negative, e.g., *k*^−3^). The spectrum of passive tracer variance, with no injection or removal and initially spread over a region large relative to the eddy scale, will depend on the spectrum of the velocity field that is stirring it. Mesoscale flows with little variance at submesoscales (thus a steep spectrum) will tend to produce tracer variance spectrum with a slope near *k*^−1^—this is the well-known Batchelor cascade^51,52^. Counter-intuitively, a velocity field with a great deal of submesoscale variability (Box 2) will produce a tracer field with relatively *less* submesoscale variance than a mesoscale velocity field would, resulting in a steeper tracer variance spectrum with slopes closer to *k*^−2^ (e.g., refs. ^53,54^). Thus, under the assumption of weak biological reactions, spectral slopes of the variance of biological tracers around *k*^−1^ could be indicative of stirring by mesoscale flows, while slopes around *k*^−2^ might indicate local stirring by submesoscale flows.

Spatial spectra of phytoplankton have been generated from data gathered through 1D in situ transects, 2D satellite ocean color data, or model results, over length scales ranging from meters to 100s of kilometers^1,55,56^. Phytoplankton spectra are often found to exhibit power-law scaling, with spectral slopes between *k*^−2^ and *k*^−1^ over mesoscale to submesoscale length scales (e.g., refs. ^1,56–60^), suggesting that passive stirring can create submesoscale phytoplankton variability by deforming large-scale regional gradients^3,61^. This is possible because the persistence time scale of phytoplankton surface blooms, 3–6 months on average^61^, is similar to the physical time over which stirring transfers variance to small scales^62^. A complexity, however, is that phytoplankton variability can also be created at small scales through active and reactive processes, yet still exhibit power-law scaling of spatial variance^49,50,63^. This complexity is reflected, for instance, in the strong temporal and regional variability of spectral slopes of ocean color variance^64^; assigning particular mechanisms to specific values of plankton spectral slopes is therefore often ill-advised. Moreover, an important caveat is that the spectrum alone does not contain information about the coherence of the underlying patchiness (Fig. 1). It is the phase relationships among the Fourier components of the spectrum that determine the presence of coherent structures—patches—of properties, such as chlorophyll concentration in the ocean^65,66^. Identical spectra can give a very patchy distribution, or a rather diffuse distribution of the property (Fig. 1), depending on the relative phases of the Fourier components.

### Subsurface stirring forms subsurface phytoplankton layers

The introduction of new instruments and sampling platforms, such as towed undulating vehicles (e.g., SeaSoar), gliders, and rapid CTD profilers (e.g., Moving Vessel Profiler) has begun to reveal the richness of the three-dimensional structure of biological properties in situ^6,67,68^. For instance, undulating glider transects in the vicinity of the intense upwelling located off the southern coast of Peru^69^ revealed several submesoscale tongues of high-salinity, high-fluorescence water extending downward from the euphotic zone (Fig. 3). The coincidence of the salinity and fluorescence supports the notion of a passive stirring mechanism in forming the layers. When plotted using density as the vertical axis, it becomes clear that these layers extend vertically across isopycnals, and tilt horizontally, consistent with the tilting and stretching of existing patches by a cross-frontal vertical shear^70^. These phytoplankton layers formed patches whose edges were well defined by their spatial gradients (Fig. 3); the patches had 2–10 km horizontal scales across the front, and persisted for weeks. The presence of high fluorescence in these layers well below the euphotic zone suggests that they originated near the surface, and became subducted at the front.

Though the orientation and vertical gradients of chlorophyll in the submesoscale patches observed by Pietri et al.^69^ suggested an origin in the euphotic zone, submesoscale subsurface patches have also been observed originating well below the surface. The formation of a subsurface thin layer, far example, was inferred from an initial phytoplankton/salinity patch at ~125 m depth, with cross-patch and along-patch horizontal scales of ~100 m and 75 km, respectively^71^. Vertical shearing of the horizontal patch gradients by low-frequency internal waves displaced the top and bottom of the patch relative to each other, forming a layer that tilted and thinned from 25 to 12 m over the course of ~20 h. Though the origin of this low-salinity/high-fluorescence layer is unknown, it is clear that stirring and shearing by the ambient flow created submesoscale horizontal and vertical structure from larger-scale gradients of the original phytoplankton patch^72^.

It is generally difficult to tease apart the relative contributions of along isopycnal stirring vs. purely horizontal stirring in forming subsurface patches. As with horizontal stirring, by deforming larger-scale gradients, the downward branch of the circulation at submesoscale dynamic fronts (Box 2) will create smaller-scale vertical structure in the tracer^40^. These downward motions will deform tracer gradients—temperature, salinity, nitrate, and phytoplankton—generating interleaved layers. Such interleaved layers in cross-frontal sections, with slopes often steeper than those of isopycnals^73–76^, can also form through horizontal deformations of existing plankton patches that extend subsurface, with no need to invoke vertical velocities.

## Reactive processes drive changes in community structure

While submesoscale phytoplankton patches can be formed by active increases in growth rate resulting from locally favorable environmental conditions, or passive stirring of existing gradients, not all members of the community respond identically to the underlying physical forcing. We would thus expect to observe altered community structure. These reactions could result from differential growth rates or different behaviors (swimming, sinking, or floating) within the phytoplankton community, or from gradients in mortality rates as predators respond to the phytoplankton distribution, and could thus propagate up the trophic chain to zooplankton and larger predators.

### Phytoplankton diversity is affected by active and passive processes

One useful measure of the reaction of planktonic communities to physical and biological forcings is their diversity. In terrestrial ecology, diversity is usefully divided into α-diversity and γ-diversity: α-diversity refers to the number of species within a local habitat, while γ-diversity describes the total number of species across multiple habitats in the region. In the ocean it can be difficult to define habitat and region. Here we will consider habitats to be waters that contain similar communities—typically determined by hydrographic properties (temperature, salinity), light availability, nutrient concentrations, and nutrient fluxes. For example, the colored patches in Fig. 3 delineate distinct habitats. In our context a region will usually refer to a frontal region, where different habitats can be brought together through stirring, or created locally by physical processes. For example, the ensemble of the colored patches in Fig. 3 represents a region. Thus, with the above definition, α-diversity will usually be found in submesoscale patches, while γ-diversity occurs at the mesoscale and larger.

Some studies have found that high planktonic γ-diversity in phytoplankton patches in frontal regions simply represents the abutment of the communities found in the water masses making up the front^48,77–79^. However, submesoscale nutrient pulses might be expected to give rise to submesoscale community patchiness (Fig. 2b, d), enhancing α-diversity and γ-diversity over that expected from purely passive stirring of regional communities, with consequences for trophic dynamics, and export fluxes. Short-term (days) responses of the nutrient-limited planktonic community to these nutrient pulses often include elevated phytoplankton growth and grazing rates, and a shift of the community toward dominance by larger phytoplankton—usually diatoms. Many studies have indeed found unique planktonic communities in submesoscale patches at fronts^6,77–82^. Distinct coastal and oceanic cyanobacterial communities, for example, were separated by a front in the Southern California Current System^81^, with the cyanobacterium *Synechococcus* dominating in the colder mesotrophic waters, and *Prochlorococcus* dominating the oligotrophic offshore waters. However, a patch with a unique phytoplankton community dominated by diatoms was found in the frontal waters. This community of large phytoplankton was coincident with increased local nitrate fluxes and decreased microzooplankton grazing^83^—all indicative of physiological and trophic responses to the frontal physical dynamics that enhanced the γ-diversity through the creation of a new, submesoscale frontal habitat.

**Fig. 6:**
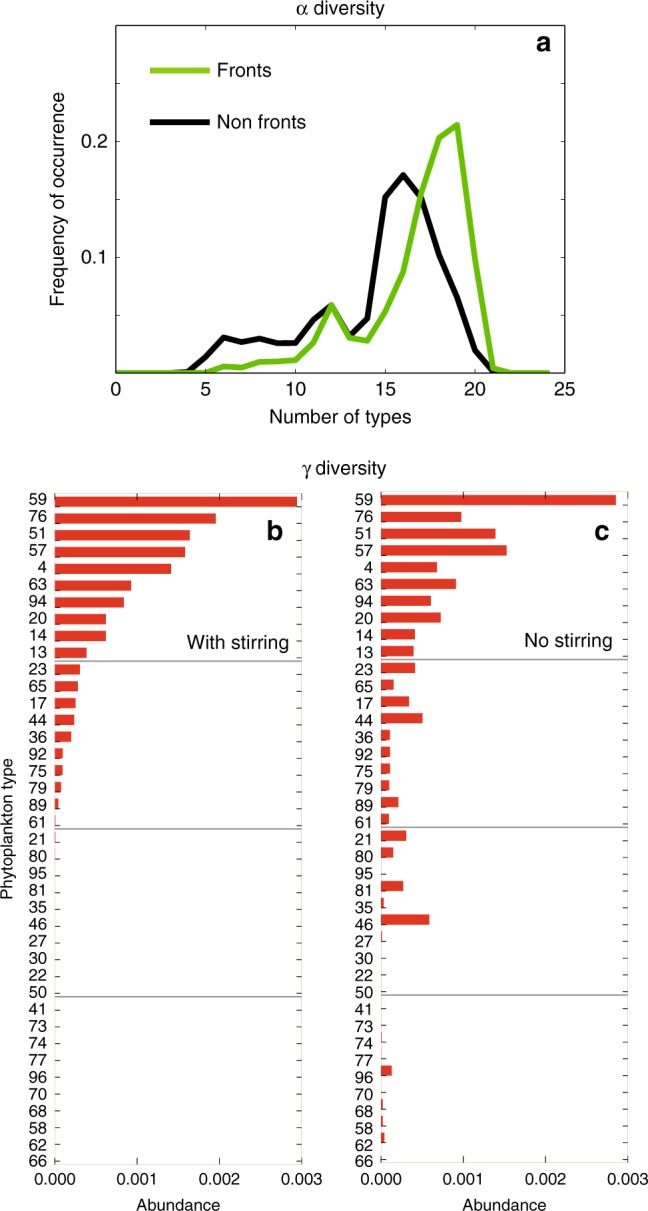
α-plankton and γ-plankton diversity. **a** The distribution of phytoplankton α-diversity over fronts (green) and away from fronts (black). Higher number of types demonstrates the increased coexistence of phytoplankton at fronts. Shown are outputs from the Darwin model comprising 100 phytoplankton types embedded in the high-resolution submesoscale flow shown in Box 3. In this model, the community structure is not imposed; rather it “self-assembles” according to the relative fitness of the phytoplankton types. The consequence is that at each time step, each model grid point is characterized by a specific community of plankton and α-diversity. **b** The abundances of the phytoplankton types that emerged over the entire region with the same simulation (with stirring) and **c** in a simulation without stirring. Stirring was suppressed by embedding the phytoplankton model in the annual time-mean flow instead of the submesoscale flow. Without stirring, the community contained more different types with more even abundances, thus the γ-diversity was larger. Thus at a local level there were more types over fronts (more α-diversity); at the regional level the stirring decreased the number of coexisting types (less γ-diversity). Adapted from Lévy et al.^84,85^

Exploring a large number of fronts, a model including 100 phytoplankton types embedded into a submesoscale-resolving flow suggested more complex changes^84^. Overall, the model generated statistically larger α-diversity at fronts than elsewhere (Fig. 6a). However, this was not systematic, with α-diversity varying among fronts, and some fronts being even less diverse than their surrounding waters. These results showed that not all fronts were efficient at driving nutrient fluxes or allowing the exploitation of new habitats. Interestingly, while in this model the fronts emerged as α-diversity and γ-diversity hot spots, stirring could in some instances decrease γ-diversity^85^ by causing the local extinction of rare species with very specific environmental niches (Fig. 6b and c).

### Plankton behavioral response interacts with active and passive processes

Phytoplankton exhibit a variety of behaviors that allow them to move relative to the water, from vertical migration to avoid predators^86^ or to gain access to light or nutrients^87^, to floating or sinking in response to nutrient stress^88^. Phytoplankton swimming behaviors can interact with the 3D flows at submesoscale fronts to generate accumulations of plankton that form submesoscale patches^89,90^. Such patches form when horizontal velocity gradients sweep the organisms toward convergence zones, where their vertical swimming (and floating) behaviors cause them to accumulate^91^. For floating organisms, these patches form within submesoscale downwelling regions; the surface patches have subsurface extensions that form stronger horizontal gradients with stronger floating speeds^91^.

### Predator–prey interactions are affected by active and passive processes

Stirring^4^, plankton motility (e.g., diel vertical migration), diffusion^63^, and active nutrient fluxes^92^ can all decorrelate phytoplankton and zooplankton distributions and lead to nonlinear changes in the grazing of phytoplankton by zooplankton^93–95^. Similar to phytoplankton, gradients of zooplankton are often (but not always^96,97^) associated with fronts. Composite analyses of hundreds of glider transects across fronts in the California Current System, for example, showed persistently elevated acoustic backscatter (proportional to zooplankton abundance/biomass) on the cold sides of the fronts^98^. The abrupt change in acoustic backscatter would seem to be indicative of abutment of two dissimilar zooplanktonic communities at the fronts, suggestive of increased γ-diversity simply through passive stirring of distinct habitats. However, taxon-resolving sampling has shown distinct that submesoscale zooplankton communities form at fronts^96,99–101^, presumably in reaction to the frontal dynamics, indicating front-enhanced zooplankton α-diversity.

In a submesoscale patch in the North Pacific subtropical gyre, lateral stirring led to a large-scale diffusion which decreased the encounter rates of phytoplankton and zooplankton, thereby reducing the grazing pressure on the phytoplankton^102^. In general, however, the magnitude of these non-linear interaction effects remains poorly quantified; they are particularly difficult to assess from observations, requiring a synoptic description of phytoplankton and zooplankton distributions, together with spatially and temporally resolved measurements of their growth, grazing, and mortality rates^103^.

### Passive and active forcings propagate up the trophic web

The enhanced abundances of eggs, nauplii, and copepodids sometimes observed at fronts^99,100^ are indicative of locally enhanced zooplankton production, presumably in response to enhanced phytoplankton growth fueled by increased nitrate fluxes at the front^83^. Though identifying the proximal cause of the enhanced nitrate fluxes is difficult, it is clear that such forcings propagate through the ecosystem, creating distinct local communities with enhanced α-diversity and γ-diversity in the frontal region.

However, while plankton mainly drift with the ocean currents, larger organisms are motile and might be predicted to have a weaker relationship with local physical forcing. Nevertheless, thanks to the development of miniaturized animal tracking devices, associations of top predators with fronts have been reported for many taxa (for example basking sharks^104^, albacore tuna^105^, whales^106^, elephant seals^107^, frigatebirds^108,109^, gannets^110^). The reasons why predators target frontal structures remain unclear and could have a variety of origins, including foraging and entrainment effects^62^, and bioenergetics^105^. One impediment to a better understanding of how submesoscale patchiness extends up the trophic web is the lack of information on the submesoscale distribution of intermediate-level fish that prey upon zooplankton and larval fish, and are foraged by top predators. Frequency-differencing echograms have revealed spatial coherence between top predators and forage fish in the submesoscale range^111^, and open the way to a better understanding of the reactive responses of ecosystems at fronts.

Given that it would likely take days for larger organisms to find and exploit physical forcings, persistent fronts lasting weeks and months present a biologically predictable ecotone. However, given the relative transience of submesoscale events, it remains unclear whether such features would be predictable enough (in an evolutionary sense) to contribute significantly to global ocean biological dynamics.

## Discussion

Field, laboratory, and modeling studies have provided extensive evidence that submesoscale oceanic currents affect not only phytoplankton concentrations, but also plankton diversity and the entire marine trophic web. Still, the spatial scales of submesoscale phenomena pose an ongoing, observational challenge, particularly when it comes to sampling biodiversity or biogeochemical fluxes. In addition, due to computational limitations, the grid resolution of global models is too coarse to assess the contribution of submesoscale dynamics to biogeochemical budgets. Today, we can only speculate on how much these dynamics affect global biogeochemical cycles, marine productivity, and ocean biodiversity. The categorization of the processes into active, passive, and reactive used in this review, although imperfect, is intended to provide a conceptual framework to guide our speculation and will hopefully open the path for more quantitative approaches in the future. Based on our synthesis, we conclude that submesoscale dynamics might not be as important as previously thought for marine productivity, but might be more important than anticipated for plankton diversity and trophic web dynamics. We want to stress that our arguments, discussed in the following, are more qualitative than quantitative.

### Submesoscale vertical nutrient transport may not be an important term in nutrient budgets

While passive stirring mainly redistributes constituents, active dynamics have the potential to strongly affect biogeochemical fluxes. This raises the question of how effective submesoscale vertical transport processes are in mediating net fluxes of nutrients within the euphotic zone. Early modeling studies of vertical nutrient transport at fronts explored persistent fronts where vertical velocities reached hundreds of meters below the surface, bringing them well into the deep nutrient pool. Such studies suggested that fronts could make a large (20–50%) contribution to total biogeochemical fluxes^5,27,112^. In addition to this modeling bias toward deep-reaching persistent fronts, there may have been a bias toward in situ observations at such fronts because they are more predictable than submesoscale fronts.

A prevalent feature of submesoscale dynamics is their perturbation through mixed-layer instability (Box 2). These instabilities are strongest in deep mixed layers, found during winter at mid-latitude and high-latitude^32^, and their associated vertical velocities tend to confined to the mixed layer^25^. Such motions are thus strongest when phytoplankton growth could be limited by the lack of light through short residence times in the euphotic zone, and weak later in the season when growth is nutrient-limited; their contribution may be significant during the transition period at the onset of the spring bloom. While this is just one of many possible dynamical processes that characterize the submesoscale, it suggests that the combination of shallow submesoscale vertical fluxes and their phasing relative to phytoplankton growth cycles over vast open ocean oceanic regions may make them inefficient at driving reactive biological responses, and thus less important in global budgets than persistent fronts.

Quantifying the importance of vertical nutrient transport at ephemeral and persistent fronts will require better global sampling and statistics. Unfortunately, the surface detection of fronts —which is feasible at the global scale with sea-surface temperature and altimetry satellite data^113^—is not sufficient to quantitatively evaluate their potential for enhanced nutrient fluxes. Regional surveys using moored arrays, gliders, and high-resolution towed instruments, such as the OSMOSIS project^114^ have greatly improved our understanding of submesoscale processes; however, one cannot confidently extrapolate the results of a North Atlantic study to the global ocean. The large array (3800 as of today) of profiling Argo floats that populates the word’s ocean providing continuous monitoring of the surface ocean vertical structure is a first step; however, this array undersamples both mesoscale and submesoscale features.

### Passive and active processes shape planktonic biodiversity

Passive processes such as mesoscale stirring do not, in principle, change planktonic α-diversity, as habitats are simply advected and distorted by the ambient flow^48^. However, mesoscale stirring can enhance γ-diversity by bringing formerly geographically distinct habitats and communities close together at frontal regions, increasing the local number of habitats. This simple consideration has started to allow us to detect γ-diversity hot-spots using ocean color satellites^115,116^. In addition, the presence of unique communities at fronts—which are sites of enhanced submesoscale physical forcing—may also reflect the effects of submesoscale dynamics on phytoplankton biomass and community structure at fronts. Such active responses of the planktonic community to submesoscale transports will affect both α-diversity and γ-diversity by creating new habitats at the fronts. Again, because of the logistical issues mentioned earlier, most in situ evidence of patterns in α-diversity at fronts comes from measurements at persistent fronts with strong nutrient fluxes. The deployment of continuously sampling underway systems offers the potential to better explore subsurface submesoscale patchiness in planktonic α-diversity.

Investigations of the relative roles of the different processes—stirring, mixing, nutrient supply, stratification, swimming—in shaping planktonic biodiversity at the submesoscale are very preliminary, and are areas of active research. Nevertheless, two characteristics lead us to speculate that these processes play crucial roles in shaping biodiversity over much broader spatial and temporal scales than the immediate frontal region. The first characteristic is the constant submesoscale perturbation of marine ecosystems: organisms that succeed in this environment must be adapted to these rapid fluctuations. Thus the environmental variability is a key characteristic, and determinant of the community structure. Geographical variations in eddy stirring^117^ and associated horizontal diffusivities^118^ show strong contrasts over broad (>1000 km) regions, suggesting a similar scale of variability for ecosystem diversity. The second characteristic is that stirring and mixing of communities that were initially apart sets them in competition with one another. When the mixing time scale is faster than the competitive exclusion time scale, such stirring may be expected to increase α-diversity and γ-diversity. The degree to which different habitats are being constantly brought together, mixed and modified by the rapidly evolving submesoscale flow will determine the outcome of species competition over longer time and larger space scales.

### Future directions

To distinguish among active, passive, and reactive processes requires high-frequency, high-resolution, 3D sampling of physical, chemical, and biological variables. Measuring down to submesoscale resolution in the field requires significant infrastructure, such as multiple ships, gliders, or large swarms of drifting floats^54,119^.

The greatest physical oceanographic challenge is to estimate submesoscale vertical velocities. Direct measurements are difficult due to the presence of inertial motions and internal waves at similar scales. Indirect estimates rely on approximations; these provide limited insight into the vertical extent of submesoscale-driven fluxes that determines their biogeochemical effectiveness^120^. The greatest biological oceanographic challenge is to quantify community composition, physiological rates, and trophic interactions with 3D submesoscale resolution.

Because fronts are regions of strong horizontal shear, vertical transport and horizontal stirring are strongly linked in the upper ocean. Thus, through this phase relationship, passive, active, and reactive processes are highly interconnected. It follows that it is difficult to determine whether a submesoscale phytoplankton patch formed as an active response to submesoscale flow or is simply the result of stirring of a larger patch that formed in response to larger-scale processes. An improved understanding of submesoscale processes will thus rely on our ability to reconstruct the Lagrangian histories of submesoscale patches from observations that are often Eulerian^62,119,121–124^. Real-time estimates of stirring from satellite altimetry can be useful for guiding adaptive sampling strategies during ship-based field campaigns to observe the evolution of tracer fields^125^. Combined Eulerian/Lagrangian sampling schemes in the field, such as making measurements while following drifters, along with supplementary transect information^126^, can help in tracking the evolution of biological communities as they are advected away from the submesoscale physical features that formed them. The anticipated 2021 launch of the new, high-resolution Surface Water and Ocean Topography (SWOT, https://swot.cnes.fr) satellite altimeter mission may provide a significant new remote-sensing tool for resolving Lagrangian trajectories with a better resolution than current altimeters^127,128^.

The contributions of submesoscale physical forcing to marine ecosystem dynamics remain unconstrained and uncertain. We are confronted with a problem at the interface of two fields—ocean physics and ecology—that lies at the frontier of knowledge in both fields, with new interaction mechanisms still being identified and their relative importance being evaluated. The strong vertical velocities associated with submesoscale fronts in the upper ocean would appear to position them to have profound biological effects. However, the vertical penetration and seasonal phasing of such dynamics may limit their efficacy in stimulating local phytoplankton growth. The considerable developments in modeling and instrumentation that have occurred over the last decades have helped in quantifying the occurrence of submesoscale biological variability. However, we will require coordinated, well-designed interdisciplinary sampling programs to disentangle the physical and biological dynamics, and in particular, the relative contributions of mesoscale and submesoscale processes. These programs must investigate both the immediate, local biological responses to submesoscale physical forcing, as well as how such responses emerge to affect global processes, such as biogeochemical cycles and biological diversity.

## References

[1] Gower J (1980). Phytoplankton patchiness indicates the fluctuation spectrum of mesoscale oceanic structure. Nature.

[2] Olson Donald, Hitchcock Gary, Mariano Arthur, Ashjian Carin, Peng Ge, Nero Redwood, Podesta Guillermo (1994). Life on the Edge: Marine Life and Fronts. Oceanography.

[3] Longhurst, A. *Ecological Geography of the Sea* (Academic Press, San Diego, 1998).

[4] Abraham E (1998). The generation of plankton patchiness by turbulent stirring. Nature.

[5] Lévy M, Klein P, Treguier A (2001). Impact of sub-mesoscale physics on production and subduction of phytoplankton in an oligotrophic regime. J. Mar. Res..

[6] Allen J (2005). Diatom carbon export enhanced by silicate upwelling in the northeast Atlantic. Nature.

[7] Mahadevan A, D’Asaro E, Lee C, Perry MJ (2012). Eddy-driven stratification initiates North Atlantic spring phytoplankton blooms. Science.

[8] Sasaki H, Klein P, Qiu B, Sasai Y (2014). Impact of oceanic-scale interactions on the seasonal modulation of ocean dynamics by the atmosphere. Nat. Commun..

[9] McWilliams JC (2016). Submesoscale currents in the ocean. Proc. R. Soc. A.

[10] Levy M, Ferrari R, Franks PJS, Martin AP, Rivière P (2012). Bringing physics to life at the submesoscale. Geophys. Res. Lett..

[11] Mahadevan A (2016). The impact of submesoscale physics on primary productivity of plankton. Annu. Rev. Mar. Sci..

[12] Shulman I (2015). Impact of submesoscale processes on dynamics of phytoplankton filaments. J. Geophys. Res. Ocean.

[13] Stolte W, McCollin T, Noordeloos AAM, Riegman R (1994). Effect of nitrogen source on the size distribution within marine phytoplankton populations. J. Exp. Mar. Bio. Ecol..

[14] Johnson KS, Riser SC, Karl DM (2010). Nitrate supply from deep to near-surface waters of the North Pacific subtropical gyre. Nature.

[15] Li QP, Franks PJS, Ohman MD, Landry MR (2012). Enhanced nitrate fluxes and biological processes at a frontal zone in the southern California current system. J. Plankton. Res..

[16] Ascani François, Richards Kelvin J., Firing Eric, Grant Scott, Johnson Kenneth S., Jia Yanli, Lukas Roger, Karl David M. (2013). Physical and biological controls of nitrate concentrations in the upper subtropical North Pacific Ocean. Deep Sea Research Part II: Topical Studies in Oceanography.

[17] Pasqueron de Fommervault (2015). Seasonal variability of nutrient concentrations in the Mediterranean Sea: contribution of bio-argo floats. J. Geophys. Res. Ocean.

[18] Bosse A (2017). A submesoscale coherent vortex in the Ligurian Sea: from dynamical barriers to biological implications. J. Geophys. Res. Ocean.

[19] Mouriño B, Fernandez E, Alves M (2004). Thermohaline structure, ageostrophic vertical velocity fields and phytoplankton distribution and production in the northeast Atlantic subtropical front. J. Geophys. Res. Ocean.

[20] Calil PHR, Richards KJ (2010). Transient upwelling hot spots in the oligotrophic North Pacific. J. Geophys. Res. Ocean.

[21] Lehahn Y, d’Ovidio F, Levy M, Heifetz E (2007). Stirring of the northeast Atlantic spring bloom: a Lagrangian analysis based on multisatellite data. J. Geophys. Res. Ocean.

[22] Liu X, Levine NM (2016). Enhancement of phytoplankton chlorophyll by submesoscale frontal dynamics in the North Pacific Subtropical Gyre. Geophys. Res. Lett..

[23] Ramachandran S, Tandon A, Mahadevan A (2014). Enhancement in vertical fluxes at a front by mesoscale–submesoscale coupling. J. Geophys. Res. Ocean.

[24] Thomas LN, Taylor JR, Ferrari R, Joyce TM (2013). Symmetric instability in the Gulf Stream. Deep Sea Res. II.

[25] Capet X, Roullet G, Klein P (2016). Intensification of upper-ocean submesoscale turbulence through Charney Baroclinic instability. J. Phys. Ocean..

[26] Lévy M., Iovino D., Resplandy L., Klein P., Madec G., Tréguier A.-M., Masson S., Takahashi K. (2012). Large-scale impacts of submesoscale dynamics on phytoplankton: Local and remote effects. Ocean Modelling.

[27] Mahadevan A, Archer D (2000). Modeling the impact of fronts and mesoscale circulation on the nutrient supply and biogeochemistry of the upper ocean. J. Geophys. Res. Ocean.

[28] McGillicuddy DJ, Anderson LA, Doney SC, Maltrud ME (2003). Eddy-driven sources and sinks of nutrients in the upper ocean: results from a 0.1° resolution model of the NorthAtlantic. Glob. Biogeochem. Cyc..

[29] Oschlies A (2002). Can eddies make ocean deserts bloom. Glob. Biogeochem. Cyc..

[30] Rosso I, Hogg AM, Matear R, Strutton PG (2016). Quantifying the influence of sub-mesoscale dynamics on the supply of iron to Southern Ocean phytoplankton blooms. Deep Sea Res. I.

[31] Lévy M (2010). Modifications of gyre circulation by sub-mesoscale physics. Ocean Model..

[32] Callies J, Ferrari R, Klymak JM, Gula J (2015). Seasonality in submesoscale turbulence. Nat. Commun..

[33] Capet, X., Campos, E. J. & Paiva, A. M. Submesoscale activity over the Argentinian shelf. *Geophys. Res. Lett*. **35**, L15605 (2008).

[34] Franks P, Walstad LJ (1997). Phytoplankton patches at fronts: a model of formation and response to wind events. J. Mar. Res..

[35] Levy, M., Klein, P. & Ben Jelloul, M. New production stimulated by high-frequency winds in a turbulent mesoscale eddy field. *Geophys. Res. Lett.* **36**, L16603 (2009).

[36] Whitt DB, Taylor JR, Lévy M (2017). Synoptic-to-planetary scale wind variability enhances phytoplankton biomass at ocean fronts. J. Geophys. Res. Ocean.

[37] Whitt DB, Lévy M, Taylor JR (2017). Low-frequency and high-frequency oscillatory winds synergistically enhance nutrient entrainment and phytoplankton at fronts. J. Geophys. Res. Ocean.

[38] Capet X, McWilliams JC, Molemaker MJ, Shchepetkin AF (2008). Mesoscale to submesoscale transition in the California Current System. Part III: energy balance and flux. J. Phys. Oceanogr..

[39] Lathuiliere C., Levy M., Echevin V. (2010). Impact of eddy-driven vertical fluxes on phytoplankton abundance in the euphotic layer. Journal of Plankton Research.

[40] Lathuilière C, Echevin V, Lévy M, Madec G (2010). On the role of the mesoscale circulation on an idealized coastal upwelling ecosystem. J. Geophys. Res. Ocean.

[41] Gruber N (2011). Eddy-induced reduction of biological production in eastern boundary upwelling systems. Nat. Geosci..

[42] Omand MM (2015). Eddy-driven subduction exports particulate organic carbon from the spring bloom. Science.

[43] Ferrari R, Merrifield ST, Taylor JR (2015). Shutdown of convection triggers increase of surface chlorophyll. JMS.

[44] Lévy M, Mémery L, Madec G (1998). The onset of a bloom after deep winter convection in the North Western Mediterranean sea: mesoscale process study with a primitive equation model. J. Mar. Syst..

[45] Taylor JR, Ferrari R (2011). Ocean fronts trigger high latitude phytoplankton blooms. Geophys. Res. Lett..

[46] Lévy, M., Gavart, M., Mémery, L., Caniaux, G. & Paci, A. A four-dimensional mesoscale map of the spring bloom in the northeast Atlantic (POMME experiment): results of a prognostic model. *J. Geophys. Res. Ocean* **110**, C07S21 (2005).

[47] Karleskind P, Lévy M, Memery L (2011). Modifications of mode water properties by sub-mesoscales in a bio-physical model of the Northeast Atlantic. Ocean Model..

[48] d’Ovidio F, De Monte S, Alvain S, Dandonneau Y, Lévy M (2010). Fluid dynamical niches of phytoplankton types. Proc. Natl Acad. Sci. USA.

[49] Powell TM, Okubo A (1994). Turbulence, diffusion and patchiness in the sea. Philos. Trans. R. Soc. Lond. B: Biol. Sci..

[50] Martin A (2003). Phytoplankton patchiness: the role of lateral stirring and mixing. Prog. Oceanogr..

[51] Batchelor GK (1959). Small-scale variation of convected quantities like temperature in turbulent fluid Part 1. General discussion and the case of small conductivity. J. Fluid Mech..

[52] Vallis, G. K. *Atmospheric and Oceanic Fluid Dynamics* (Cambridge University Press, Cambridge, 2006).

[53] Keating SR, Smith KS, Kramer PR (2011). Diagnosing lateral mixing in the upper ocean with virtual tracers: spatial and temporal resolution dependence. J. Phys. Oceanogr..

[54] Poje AC (2014). Submesoscale dispersion in the vicinity of the Deepwater Horizon spill. Proc. Natl Acad. Sci. USA.

[55] Mackas DL, Boyd CM (1979). Spectral analysis of zooplankton spatial heterogeneity. Science.

[56] Martin AP, Srokosz MA (2002). Plankton distribution spectra: inter‐size class variability and the relative slopes for phytoplankton and zooplankton. Geophys. Res. Lett..

[57] Yoder JA, Aiken J, Swift RN, Hoge FE, Stegmann PM (1993). Spatial variability in near-surface chlorophyll a fluorescence measured by the Airborne Oceanographic Lidar (AOL). Deep Sea Res. II.

[58] Washburn L, Emery BM, Jones BH, Ondercin DG (1998). Eddy stirring and phytoplankton patchiness in the subarctic North Atlantic in late summer. Deep Sea Res. I.

[59] Piontkovski SA, Williams R, Peterson WT (1997). Spatial heterogeneity of the planktonic fields in the upper mixed layer of the open ocean. Mar. Ecol. Prog. Ser..

[60] Van Gennip S (2016). Plankton patchiness investigated using simultaneous nitrate and chlorophyll observations. J. Geophys. Res. Ocean.

[61] Demarcq H, Reygondeau G, Alvain S, Vantrepotte V (2012). Monitoring marine phytoplankton seasonality from space. Remote Sens. Environ..

[62] Lehahn Y, d’Ovidio F, Koren I (2017). A satellite-based Lagrangian view on phytoplankton dynamics. Annu. Rev. Mar. Sci..

[63] Bracco A, Clayton S, Pasquero C (2009). Horizontal advection, diffusion, and plankton spectra at the sea surface. J. Geophys. Res. Ocean.

[64] Van Gennip, S. J. *Understanding the Extent of Universality in Phytoplankton Spatial Properties*. Ph.D. thesis, Univ. Southampton, 1–176 (2015).

[65] Armi L, Flament P (1985). Cautionary remarks on the spectral interpretation of turbulent flows. J. Geophys. Res. Ocean.

[66] McWilliams JC (1984). The emergence of isolated coherent vortices in turbulent flow. J. Fluid Mech..

[67] Nurser A, Zhang J (2000). Eddy-induced mixed layer shallowing and mixed layer/thermocline exchange. J. Geophys. Res. Ocean.

[68] Niewiadomska K, Claustre H, Prieur L, D’Ortenzio F (2008). Submesoscale physical-biogeochemical coupling across the Ligurian current (northwestern Mediterranean) using a bio-optical glider. Limnol. Oceanogr..

[69] Piétri A (2013). Finescale vertical structure of the upwelling system off Southern Peru as observed from glider data. J. Phys. Oceanogr..

[70] Birch DA, Young WR, Franks PJS (2008). Thin layers of plankton: formation by shear and death by diffusion. Deep Sea Res. I.

[71] Hodges BA, Fratantoni DM (2009). A thin layer of phytoplankton observed in the Philippine Sea with a synthetic moored array of autonomous gliders. J. Geophys. Res. Ocean.

[72] Durham WM, Stocker R (2012). Thin phytoplankton layers: characteristics, mechanisms, and consequences. Annu. Rev. Mar. Sci..

[73] Macvean MK, Woods JD (1980). Redistribution of scalars during upper ocean frontogenesis: a numerical model. Q. J. R. Meteorol. Soc..

[74] Klein P (1998). Three-dimensional stirring of thermohaline fronts. J. Mar. Res..

[75] Smith KS, Ferrari R (2009). The production and dissipation of compensated thermohaline variance by mesoscale stirring. J. Phys. Oceanogr..

[76] Shcherbina AY, Gregg MC, Alford MH, Harcourt RR (2010). Three-dimensional structure and temporal evolution of submesoscale thermohaline intrusions in the North Pacific Subtropical Frontal Zone. J. Phys. Oceanogr..

[77] Clayton S, Nagai T, Follows MJ (2014). Fine scale phytoplankton community structure across the Kuroshio Front. J. Plankton Res..

[78] Clayton S, Lin YC, Follows MJ, Worden AZ (2016). Co-existence of distinct Ostreococcusecotypes at an oceanic front. Limnol. Oceanogr..

[79] Mousing Erik Askov, Richardson Katherine, Bendtsen Jørgen, Cetinić Ivona, Perry Mary Jane (2016). Evidence of small-scale spatial structuring of phytoplankton alpha- and beta-diversity in the open ocean. Journal of Ecology.

[80] Claustre H (1994). Phytoplankton dynamics associated with a geostrophic front: ecological and biogeochemical implications. J. Mar. Res..

[81] Taylor AG (2012). Sharp gradients in phytoplankton community structure across a frontal zone in the California Current Ecosystem. J. Plankton Res..

[82] Cetinić I (2015). A simple optical index shows spatial and temporal heterogeneity in phytoplankton community composition during the 2008 North Atlantic Bloom Experiment. Biogeosciences.

[83] Li QP, Franks PJS, Ohman MD, Landry MR (2012). Enhanced nitrate fluxes and biological processes at a frontal zone in the southern California current system. J. Plankton Res..

[84] Lévy Marina, Jahn Oliver, Dutkiewicz Stephanie, Follows Michael J., d'Ovidio Francesco (2015). The dynamical landscape of marine phytoplankton diversity. Journal of The Royal Society Interface.

[85] Lévy M, Jahn O, Dutkiewicz S, Follows MJ (2014). Phytoplankton diversity and community structure affected by oceanic dispersal and mesoscale turbulence. Limnol. Oceanogr.: Fluids Env..

[86] Bollens SM, Quenette JA, Rollwagen-Bollens G (2012). Predator-enhanced diel vertical migration in a planktonic dinoflagellate. Mar. Ecol. Prog. Ser..

[87] Jephson T, Carlsson P (2009). Species- and stratification-dependent diel vertical migration behaviour of three dinoflagellate species in a laboratory study. J. Plankton Res..

[88] Gemmell BJ, Oh G, Buskey EJ, Villareal TA (2016). Dynamic sinking behaviour in marine phytoplankton: rapid changes in buoyancy may aid in nutrient uptake. Proc. Biol. Sci..

[89] Franks, P. Phytoplankton blooms at fronts: patterns, scales, and physical forcing mechanisms. *Rev. Aquat. Sci.* **6**, 121–137 (1992).

[90] Flierl GR, Woods NW (2015). Copepod aggregations: influences of physics and collective behavior. J. Stat. Phys..

[91] Taylor JR (2018). Accumulation and subduction of buoyant material at submesoscale fronts. J. Phys. Oceanogr..

[92] Mahadevan A, Campbell JW (2002). Biogeochemical patchiness at the sea surface. Geophys. Res. Lett..

[93] Wallhead PJ, Martin AP, Srokosz MA (2008). Spatially implicit plankton population models: transient spatial variability. J. Theor. Biol..

[94] Lévy M, Martin AP (2013). The influence of mesoscale and submesoscale heterogeneity on ocean biogeochemical reactions. Glob. Biogeochem. Cycles.

[95] Neufeld Z (2012). Stirring effects in models of oceanic plankton populations. Chaos.

[96] Molinero JC, Ibanez F, Souissi S, Bosc E, Nival P (2008). Surface patterns of zooplankton spatial variability detected by high frequency sampling in the NW Mediterranean. Role of density fronts. JMS.

[97] Luo JY (2014). Environmental drivers of the fine-scale distribution of a gelatinous zooplankton community across a mesoscale front. Mar. Ecol. Prog. Ser..

[98] Powell JR, Ohman MD (2015). Changes in zooplankton habitat, behavior, and acoustic scattering characteristics across glider-resolved fronts in the Southern California Current System. Prog. Oceanogr..

[99] Ohman MD, Powell JR, Picheral M, Jensen DW (2012). Mesozooplankton and particulate matter responses to a deep-water frontal system in the southern California Current System. J. Plankton Res..

[100] Lane P, Smith SL, Graber HC (2003). Mesoscale circulation and the surface distribution of copepods near the south Florida Keys. Bull. Mar. Sci..

[101] Greer AT, Cowen RK, Guigand CM, Hare JA (2015). Fine-scale planktonic habitat partitioning at a shelf-slope front revealed by a high-resolution imaging system. JMS.

[102] Lehahn Y (2017). Dispersion/dilution enhances phytoplankton blooms in low-nutrient waters. Nat. Commun..

[103] Martin AP (2015). An observational assessment of the influence of mesoscale and submesoscale heterogeneity on ocean biogeochemical reactions. Glob. Biogeochem. Cyc..

[104] Sims DW, Quayle VA (1998). Selective foraging behaviour of basking sharks on zooplankton in a small-scale front. Nature.

[105] Snyder S, Franks PJS, Talley LD, Xu Y, Kohin S (2017). Crossing the line: Tunas actively exploit submesoscale fronts to enhance foraging success. Limnol. Oceanogr..

[106] Cotté C (2011). Scale‐dependent interactions of Mediterranean whales with marine dynamics. Limnol. Oceanogr..

[107] Cotté C, d’Ovidio F, Dragon AC, Guinet C, Levy M (2015). Flexible preference of southern elephant seals for distinct mesoscale features within the Antarctic Circumpolar Current. Prog. Oceanogr..

[108] De Monte S (2012). Frigatebird behaviour at the ocean–atmosphere interface: integrating animal behaviour with multi-satellite data. J. R. Soc. Interface.

[109] Tew Kai E (2009). Top marine predators track Lagrangian coherent structures. Proc. Natl Acad. Sci. USA.

[110] Sabarros PS (2014). Fine-scale recognition and use of mesoscale fronts by foraging Cape gannets in the Benguela upwelling region. Deep Sea Res. II.

[111] Benoit-Bird KJ (2013). Prey patch patterns predict habitat use by top marine predators with diverse foraging strategies. PLoS ONE.

[112] Spall S, Richards K (2000). A numerical model of mesoscale frontal instabilities and plankton dynamics—I. Model formulation and initial experiments. Deep Sea Res. I.

[113] Prants SV, Budyansky MV, Uleysky MY (2014). Identifying Lagrangian fronts with favourable fishery conditions. Deep Sea Res. I.

[114] Thompson AF (2016). Open-ocean submesoscale motions: a full seasonal cycle of mixed layer instabilities from gliders. J. Phys. Oceanogr..

[115] De Monte S, Soccodato A, Alvain S, d’Ovidio F (2013). Can we detect oceanic biodiversity hotspots from space|[quest]|. ISME J..

[116] Soccodato A (2016). Estimating planktonic diversity through spatial dominance patterns in a model ocean. Mar. Genom..

[117] Cole Sylvia T., Wortham Cimarron, Kunze Eric, Owens W. Brechner (2015). Eddy stirring and horizontal diffusivity from Argo float observations: Geographic and depth variability. Geophysical Research Letters.

[118] Abernathey RP, Marshall J (2013). Global surface eddy diffusivities derived from satellite altimetry. J. Geophys. Res. Ocean.

[119] Jaffe JS (2017). A swarm of autonomous miniature underwater robot drifters for exploring submesoscale ocean dynamics. Nat. Commun..

[120] Giordani H, Prieur L, Caniaux G (2006). Advanced insights into sources of vertical velocity in the ocean. Ocean Dyn..

[121] Alkire MB, D’Asaro E, Lee C, Perry MJ, Gray A (2012). Estimates of net community production and export using high-resolution, Lagrangian measurements of O_2_, NO_3_, and POC through the evolution of a spring diatom bloom in the North Atlantic. Deap Sea Res..

[122] d’Ovidio F, De Monte S, Penna AD, Cotté C, Guinet C (2013). Ecological implications of eddy retention in the open ocean: a Lagrangian approach. J. Phys. A: Math. Theor..

[123] Brody SR, Lozier MS (2016). Quantifying the impact of submesoscale processes on the spring phytoplankton bloom in a turbulent upper ocean using a Lagrangian approach. Geophys. Res. Lett..

[124] de Verneil A, Franks PJS (2015). A pseudo‐Lagrangian method for remapping ocean biogeochemical tracer data: Calculation of net Chl‐a growth rates. J. Geophys. Res. Ocean.

[125] d’Ovidio F (2015). The biogeochemical structuring role of horizontal stirring: Lagrangian perspectives on iron delivery downstream of the Kerguelen Plateau. Biogeosciences.

[126] Landry MR, Ohman MD, Goericke R, Stukel MR, Tsyrklevich K (2009). Lagrangian studies of phytoplankton growth and grazing relationships in a coastal upwelling ecosystem off Southern California. Prog. Oceanogr..

[127] Fu LL, Ferrari R (2008). Observing oceanic submesoscale processes from space. Eos Trans. Am. Geophys. Union.

[128] Qiu B (2016). Reconstructability of three-dimensional upper-ocean circulation from SWOT sea surface height measurements. J. Phys. Ocean.

[129] Swallow J (1957). Some further deep current measurements using neutrally buoyant floats. Deap Sea Res..

[130] Defant, A. *Physical Oceanography* (Pergamon Press, London, 1961).

[131] MODE. (1978). The Mid-Ocean Dynamics Experiment. Deap Sea Res..

[132] Gill AE, Green JSA, Simmons AJ (1974). Energy partition in the large-scale ocean circulation and the production of mid-ocean eddies. Deap Sea Res..

[133] Fu Lee-Lueng (1983). Recent progress in the application of satellite altimetry to observing the mesoscale variability and general circulation of the oceans. Reviews of Geophysics.

[134] Stammer D, Wunsch C (1994). Preliminary assessment of the accuracy and precision of TOPEX/POSEIDON altimeter data with respect to the large scale ocean circulation. J. Geophys. Res. Oceans.

[135] Munk W, Armi L, Fischer K, Zachariasen F (2000). Spirals on the sea. Proc. R. Soc. A.

[136] Thomas L, Lee C (2005). Intensification of ocean fronts by down-front winds. J. Phys. Oceanogr..

[137] D’Asaro E, Lee C, Rainville L, Harcourt R, Thomas L (2011). Enhanced turbulence and energy dissipation at ocean fronts. Science.

[138] Thomas L, Tandon A, Mahadevan A (2008). Submesoscale processes and dynamics. Eddy Resolv. Ocean Models Geophys. Monogr..

[139] Brannigan L, Marshall DP, Naveira Garabato A, Nurser AJG (2015). The seasonal cycle of submesoscale flows. Ocean Model..

[140] Callies J, Flierl G, Ferrari R, Fox-Kemper B (2016). The role of mixed-layer instabilities in submesoscale turbulence. J. Fluid Mech..

[141] Buckingham CE (2016). Seasonality of submesoscale flows in the ocean surface boundary layer. Geophys. Res. Lett..

[142] Boccaletti G, Ferrari R, Fox-Kemper B (2007). Mixed layer instabilities and restratification. J. Phys. Oceanogr..

[143] Fox-Kemper B, Ferrari R, Hallberg R (2008). Parameterization of mixed layer eddies. Part I: theory and diagnosis. J. Phys. Oceanogr..

[144] Tulloch R, Hill C, Smith KS, Tulloch R, Marshall J (2011). Scales, growth rates, and spectral fluxes of baroclinic instability in the ocean. J. Phys. Ocean..

[145] Thomas L, Ferrari R (2008). Friction, frontogenesis, and the stratification of the surface mixed layer. J. Phys. Oceanogr..

[146] Shcherbina AY (2015). The LatMix summer campaign: submesoscale stirring in the upper ocean. Bull. Am. Meteor. Soc..

[147] Qiu B, Chen S, Klein P, Sasaki H, Sasai Y (2014). Seasonal mesoscale and submesoscale eddy variability along the North Pacific Subtropical countercurrent. J. Phys. Oceanogr..

[148] Gent PR, McWilliams JC (1996). Eliassen-Palm fluxes and the momentum equations in non-eddy-resolving ocean circulation models. J. Phys. Oceanogr..

[149] Ferrari R (2011). A frontal challenge for climate models. Science.

[150] Lévy M (2012). Grid degradation of submesoscale resolving ocean models: benefits for offline passive tracer transport. Ocean Model..

[151] Gula J, Molemaker MJ, McWilliams JC (2014). Submesoscale cold filaments in the Gulf Stream. J. Phys. Oceanogr..

